# Unclassified complex urogenital anomaly in a 3-year-old girl: Diagnostic and surgical challenges

**DOI:** 10.1016/j.eucr.2026.103379

**Published:** 2026-02-17

**Authors:** Ilaria Manghi, Tommaso Gargano, Francesca Ruspi, Michela Maffi, Michele Libri, Marcello Domini

**Affiliations:** Pediatric Surgery Department, IRCCS Sant'Orsola-Malpighi Polyclinic, Alma Mater Studiorum-University of Bologna, 40126, Bologna, Italy

**Keywords:** Complex urogenital malformations, Disorder of sex development (DSD), Neovagina

## Abstract

Complex urogenital malformations are rare congenital anomalies that often escape existing classification systems due to marked anatomical variability. We describe a previously unreported urogenital malformation.

A 3-year-old girl presented with ambiguous genitalia, a single perineal urogenital orifice, bilateral duplicated renal systems with complete vesicoureteral reflux, and vaginal agenesis. Endoscopy revealed a bladder dome diverticulum containing ureteral and cervical orifices. She underwent bilateral ureteral reimplantation and creation of an ileal neovagina with utero-neovaginal anastomosis, with an uneventful postoperative course.

This anomaly represents a previously unreported entity and underscores the need for individualized, multidisciplinary management.

## 1 Introduction

Complex genitourinary anomalies represent exceptionally rare developmental defects arising from the close embryologic relationship between the genital and urinary systems. During early embryogenesis, both systems share a common origin from the intermediate mesoderm, and by the end of the ninth gestational week, the paramesonephric ducts project into the dorsal wall of the urogenital sinus as the Müllerian tubercle, from which the vaginal plate develops.^1^ Over the years, several classification systems have been proposed,^2^ yet none fully capture the broad spectrum of possible malformations. Most frameworks, such as the ASRM classification^3^ for Müllerian duct anomalies and Hendren's system for urogenital sinus defects,^4^ tend to separate anomalies by organ system. As a result, combined genitourinary malformations often fall outside these categories. We describe the case of a 3-year-old girl with a common urogenital channel opening into the bladder dome, where three distinct orifices were observed—two corresponding to the ureteral meatuses and one to the uterine cervix. To our knowledge, no similar case has been reported. This unique anatomy posed both diagnostic and therapeutic challenges, emphasizing the limits of current classifications and the importance of a multidisciplinary approach in managing such rare conditions.

This manuscript was prepared following the CARE guidelines.

## 2 Case report

**Fig. 1:**
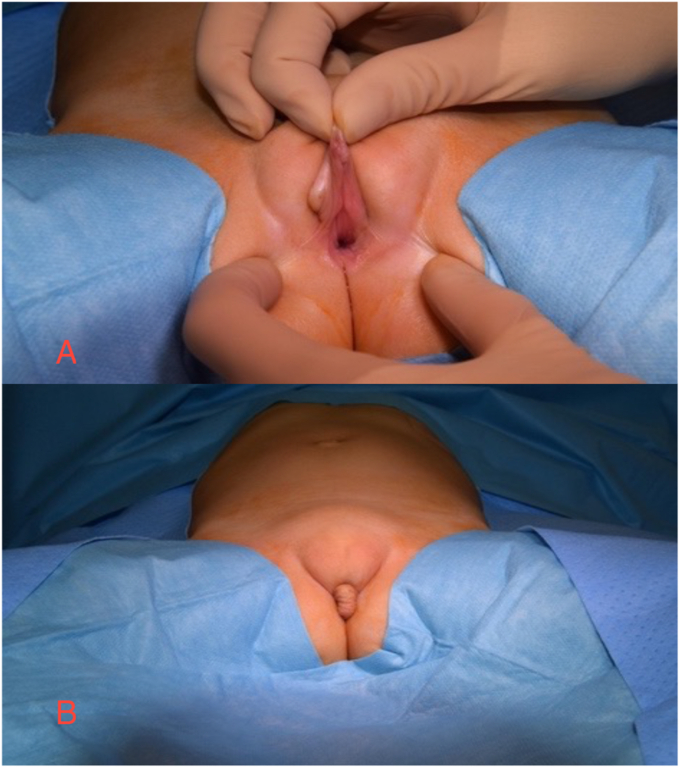
Appearance of the external genitalia A) presence of single genitourinary orifice, B) cutaneous flap.

We report the case of a patient referred to our center at 3 years of age, born with a prenatal suspicion of Disorder of Sex Development (DSD) due to the appearance of ambiguous genitalia, specifically a cutaneous flap mimicking a pseudophallus. This suspicion was confirmed at birth: physical examination revealed a normally positioned anus and, anteriorly, a single wide urogenital orifice (Fig. 1). Partial labial fusion and a cutaneous flap resembling a prepuce were also noted. At her local hospital, the patient underwent cystoscopy and catheterization, which were continued until presentation at our center, due to persistent continuous urinary incontinence. Her medical history included two episodes of pyelonephritis since which she had been maintained on antibiotic prophylaxis.

Upon admission, the patient underwent an extensive diagnostic work-up, including laboratory tests and imaging studies. No associated cardiac, skeletal or neurological anomalies were detected. Abdominal ultrasound demonstrated diffusely increased parenchymal echogenicity with poor corticomedullary differentiation in both kidneys. Two small cortical cystic lesions were observed in the right kidney, with mild ectasia (2–3 mm) of the sub-pyelic ureter but no significant dilatation of the calyceal-pelvic system. The left kidney showed two distinct portions: an upper portion with markedly increased echogenicity and poor corticomedullary differentiation, and a lower portion with relatively preserved echotexture; no significant excretory system dilatation was noted.

Cystourethrography revealed complete bilateral vesicoureteral reflux with pyelointerstitial reflux during voiding, as well as a bifid appearance suggestive of an incomplete duplicated collecting system. This finding was subsequently confirmed by uro-CT, which demonstrated bilateral ectopic ureteral insertion and a diverticular outpouching at the bladder dome. The uterus and adnexal structures were not clearly visualized. DMSA renal scintigraphy showed mild asymmetry in renal size, with the right kidney slightly smaller, but cortical function was preserved in the upper portion of the right duplicated system, and no clear evidence of scarring was observed.

**Fig. 2:**
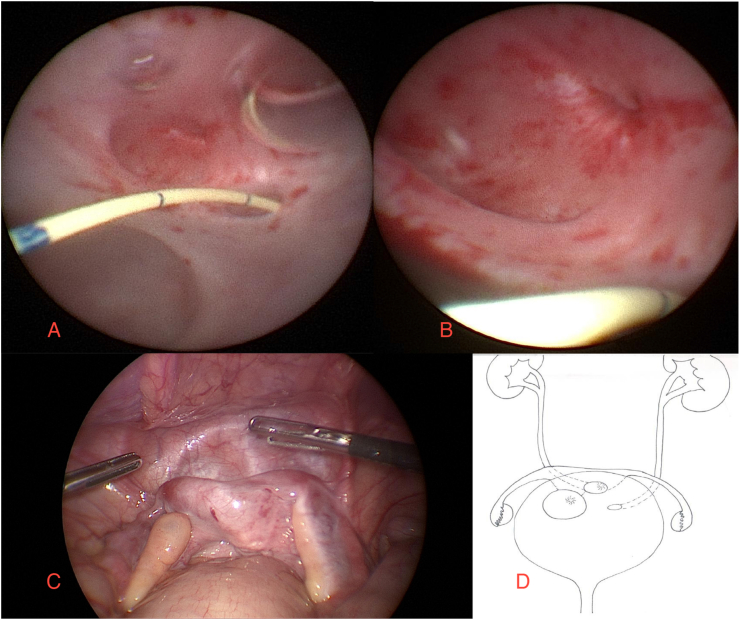
A, B: Cystoscopic view showing three distinct orifices: two ureteral meatuses and the uterine cervix. C: Laparoscopic view of bicornute uterus and two ovaries. D: Schematic illustration of the patient's urogenital anatomy.

A multidisciplinary evaluation involving genetics and endocrinology was conducted. The patient then underwent combined diagnostic laparoscopy and cystoscopy (Fig. 2). The urethral canal appeared wide, while the bladder neck was poorly represented. On the bladder dome, a diverticulum and three distinct orifices were identified, one resembling a cervix. Laparoscopic biopsies revealed prepubertal ovarian cortical tissue with primordial follicles bilaterally. Concurrent genetic analysis confirmed a 46, XX karyotype.

**Fig. 3:**
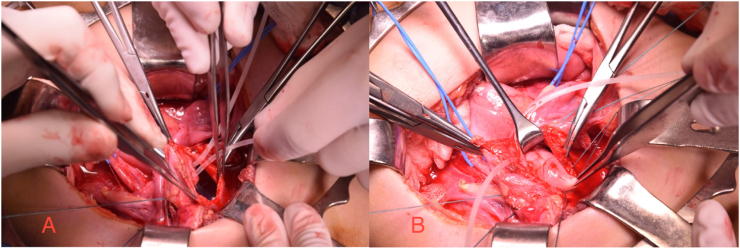
Intraoperative view: A) ureteral reimplantation: ureteral cannulation; B) isolation of uterine cervix.

Following a multidisciplinary discussion involving radiologists, pediatric endocrinologists, and pediatric surgeons, the patient underwent bilateral ureteral reimplantation using the Politano technique, along with creation of a neovagina using an ileal segment with ileoileal and utero-neovaginal anastomosis (Fig. 3). The postoperative course was uneventful, and the patient was discharged on postoperative day 16. Examination under anesthesia at discharge confirmed an elastic, normally calibrated vaginal anastomosis. At current follow-up, no urinary tract infections or other complications have been reported.

At the time of discharge, the patient exhibited persistent urinary incontinence. Nevertheless, a staged surgical intervention at the level of the bladder neck is planned to enhance bladder outlet resistance and achieve urinary continence. A comprehensive invasive urodynamic evaluation will be performed in close temporal proximity to the procedure to accurately assess bladder capacity and lower urinary tract function, thereby optimizing surgical planning and strategy. Regarding the external genital appearance, no surgical procedure was performed to correct the cosmetic aspect, given the young age of the patient.

## 3 Discussion

We presented this clinical case because of the uniqueness of the malformation. During the diagnostic process, an extensive literature search was conducted to identify cases comparable to that of our patient.

As discussed in the introduction, the rarity and heterogeneity of complex urogenital malformations make their inclusion within existing classification systems particularly challenging. Classifications are meant to simplify the malformative spectrum, yet such complex anomalies often defy these frameworks.

No case reports describing a malformation identical to our patient's condition were found in the literature. However, analogies with other rare presentations were identified, which helped us define the most appropriate surgical approach.

One comparable case^5^ involved a 15-year-old girl presenting with *menouria*, in whom cystoscopy revealed a vesicouterine fistula associated with a bicornuate uterus, vaginal agenesis, and renal agenesis. Unlike our patient, the bladder trigone was normally represented, and no disorders of sexual differentiation or external genital anomalies were reported. Surgical treatment in that case involved the creation of a neovagina using an ileal flap (Monti technique) anastomosed to the uterine cervix.

Genitourinary fistulas^6^ are rare and, in most cases, are acquired—typically following cesarean section, hysterectomy, colorectal surgery, radiotherapy, or gender-based violence. Congenital genitourinary fistulas, on the other hand, are exceptional. According to Jóźwik et al.,^7^ in a meta-analysis of ten reported cases of congenital vesicouterine fistula, there was a strong correlation with renal and vaginal agenesis—unlike our patient, who presented with complete vaginal agenesis but only bilateral duplex renal system.

In none of the reported cases of vesicouterine fistula were disorders of sexual differentiation (DSD) described. Therefore, alternative hypotheses were considered, including a rare form of high urogenital sinus,^8^ in which the vaginal canal and ureters open at the level of the bladder trigone. In such cases, the external genitalia may appear ambiguous, with the presence of a pseudophallus. Similar features may also be observed in posterior cloaca, particularly in type A,^9^ where the urogenital sinus opens perineally, anterior to the anus and rectum, which—unlike other types of anorectal malformations—remains normally developed.

As with other congenital anomalies, posterior cloaca^10^ represents a spectrum of malformations, ranging from mild posterior deviation of a short or ultra-short common urogenital sinus to severe posterior displacement of the urogenital tract, often associated with a characteristic *“doll-like” perineum*. In the most severe forms, this may occur with or without complete absence of the urinary bladder, where total urinary incontinence is an expected finding.

It is therefore possible to draw some anatomical parallels between our patient's presentation and those described in posterior cloaca; however, her condition remains distinct and not fully comparable to any previously reported case.

From a surgical standpoint, the separation of the genital and urinary systems was mandatory to prevent *menouria* at puberty and risk of peritonitis due to the passage of urine in the peritoneum through the fallopian tubes. The ectopic ureteral insertion and resulting intrarenal reflux required a bilateral ureteroneocystostomy. The separation of the bicornuate uterus from the bladder dome necessitated reconstruction and the creation of a neovagina using an ileal segment. No surgical correction of the external genitalia was performed, as this will be deferred until the patient reaches an age at which she can participate in the decision-making process regarding any aesthetic or reconstructive procedures.

## 4 Conclusions

The case described has no equivalent in the existing literature. A thorough analysis of possible analogies—based on both the anatomical presentation and the embryologic development of the defect—suggests similarities with entities such as vesicouterine fistula, posterior cloaca, and high urogenital sinus.

Nevertheless, none of these malformations fully account for the unique clinical picture observed in our patient. This raises the possibility that we are facing either a previously unreported malformation arising from a single embryologic error, or a rare combination of multiple developmental anomalies.

This case highlights the complexity of urogenital embryogenesis and underscores the importance of a multidisciplinary approach in both diagnosis and surgical management of such exceptional condition.

## CRediT authorship contribution statement

**Ilaria Manghi:** Writing – original draft, Project administration, Data curation, Conceptualization. **Tommaso Gargano:** Writing – review & editing, Supervision. **Francesca Ruspi:** Writing – original draft. **Michela Maffi:** Writing – review & editing, Supervision. **Michele Libri:** Writing – review & editing, Supervision. **Marcello Domini:** Writing – review & editing, Supervision.

## Statements

All authors attest that they meet the current ICMJE criteria for Authorship.

Informed consent for the publication of this case has been obtained from the patient's parents.

## Disclosure

Nothing.
